# Supervised-actor-critic reinforcement learning for intelligent mechanical ventilation and sedative dosing in intensive care units

**DOI:** 10.1186/s12911-020-1120-5

**Published:** 2020-07-09

**Authors:** Chao Yu, Guoqi Ren, Yinzhao Dong

**Affiliations:** 1 School of Data and Computer Science, Sun Yat-Sen University, Guangzhou, 510015 China; 2 School of Computer Science and Technology, Dalian University of Technology, Dalian, 110621 China

**Keywords:** Reinforcement learning, Inverse learning, Mechanical ventilation, Sedative dosing, Intensive care units

## Abstract

**Background:**

Reinforcement learning (RL) provides a promising technique to solve complex sequential decision making problems in healthcare domains. Recent years have seen a great progress of applying RL in addressing decision-making problems in Intensive Care Units (ICUs). However, since the goal of traditional RL algorithms is to maximize a long-term reward function, exploration in the learning process may have a fatal impact on the patient. As such, a short-term goal should also be considered to keep the patient stable during the treating process.

**Methods:**

We use a Supervised-Actor-Critic (SAC) RL algorithm to address this problem by combining the long-term goal-oriented characteristics of RL with the short-term goal of supervised learning. We evaluate the differences between SAC and traditional Actor-Critic (AC) algorithms in addressing the decision making problems of ventilation and sedative dosing in ICUs.

**Results:**

Results show that SAC is much more efficient than the traditional AC algorithm in terms of convergence rate and data utilization.

**Conclusions:**

The SAC algorithm not only aims to cure patients in the long term, but also reduces the degree of deviation from the strategy applied by clinical doctors and thus improves the therapeutic effect.

## Background

In the healthcare field, a clinical treatment plan consists of a series of decisions that determine the type of treatment and the dose of drug based on the current health condition and past treatment history of a patient. Therefore, the clinical treatment is usually characterized by a sequential decision-making process that lasts for a long period. RL aims to solve this kind of sequential decision-making problems when an agent chooses an action at each time step based on its current state, and receives an evaluative feedback and the new state from the environment [1]. In the past decades, applying RL for more efficient decision-making has become a hot research topic in healthcare domains [2], generating a great breakthrough in treatment of diabetics [3], cancer [4], sepsis [5], and many other diseases [6–8].

In the clinical treatment, clinicians should achieve the long-term goal of curing the patients, but at the same time, the dosage of the medicine should be controlled in daily range so as to maintain the stable condition of patients. The short-term object is also critical as it can avoid additional risk to patients due to improper dosage. Traditional RL, however, mainly considers inherent time delay that is assessed by long-term goals, but lacks of consideration of short-term effect. This may lead to a large cumulative reward for the learned strategy, but this strategy can deviate from the clinical treatment strategy significantly. In this paper, a Supervised-Actor-Critic (SAC) [9] algorithm is applied to solve the above problem. The agent of SAC takes curing a patient as the long-term goal, and the deviation degree of the treatment between the SAC agent and the clinician as the short-term goal. An expert strategy is defined as a Supervisor, which is used to guide the learning process to reduce the additional treatment risk to patients.

In the following sections, we first introduce some recent research progress of applying RL methods in ICUs. Then, we present the data preprocessing details and formalize the decision making process of mechanical ventilation and sedative dosing in ICUs. We then introduce the SAC algorithm in detail and discuss the results and analysis between SAC and AC. Finally, we conclude this paper with future works.

## Related work

The development of artificial intelligence (AI) techniques and data processing methods enable optimal diagnose, treat and mortality prediction of patients in ICUs [10]. As one of the core AI technologies, RL has been widely applied in realizing intelligent decision-making in ICUs [2]. The authors [11–13] applied RL algorithms in addressing the administration of *intravenous* (IV) and maximum *vasopressor* (VP) in sepsis treatment. Padmanabhan et al. [14, 15] proposed an RL-based control strategy for ICU sedation regulation. Prasad et al. [16] applied fitted Q iteration with extremely randomized trees to determine the best weaning time of invasive mechanical ventilation. Utomo et al. [17] proposed a graphical model that was able to show transitions of patient health conditions and treatments for better explanability, and applied RL to generate a real-time treatment recommendation in ICUs. Nemati et al. [18] used deep RL methods to calculate optimal unfractionated Heparin from sub-optimal clinical ICU data. Yu et al. [19] used inverse RL to infer the reward functions when dealing with mechanical ventilation and sedative dosing in ICUs. Chang et al. [20] proposed a Q-learning method that jointly minimized the measurement cost and maximized predictive gain, by scheduling strategically-timed measurements in ICUs. Unlike all the existing studies that only consider the long term effects of treatment using RL methods, we also consider the short-term effects of treatment in terms of deviation from the doctor’s clinical treatment expectations, in order to ensure safety during the learning process.

## Preprocessing

### Ventilation and sedation dosing in ICUs

Effective ventilation is one of the most commonly used methods in the treatment of patients in ICUs. These patients are usually featured with acute respiratory failure or impaired lung function caused by some underlying factors, such as pneumonia, sepsis or heart disease. In addition, respiratory support is required after major surgery for consciousness disorders or weakness. Whether a patient is ready for extubation is determined by some major diagnostic tests, involving screening for potential disease resolution, hemodynamic stability, current ventilator assessment settings and awareness levels, and the final series of spontaneous breathing tests (SBTs). Serious discomfort and longer time stay in ICUs will occur if a patient must be reintubated due to the failure of breath test and other reasons within her first stay of 48 to 72 h.

In ICUs, another major treatment means is the use of sedative doses, which is essential for maintaining the patient’s physiological stability. According to a relevant research, there is a certain correlation between the timing of ventilation and the application of sedatives in ICUs. Therefore, it is necessary to propose more effective ventilation and dosing methods so as to improve the patient’s treatment effect, and reduce the patient’s residence time and associated cost in ICUs.

### Data processing

Firstly, we extract 8860 admissions from adult patients in MIMIC-III database [21], and exclude those admissions who were kept under ventilation for less than 24 hours, or failed being discharged from ICUs at the end of admission. The MIMIC is a free resource-rich ICU research database, which was first published in 2006 by the Computational Physiology Laboratory of the Massachusetts Institute of Technology (MIT), the Beth Israel Dikang Medical Center (BIDMC) and Philips Medical Center. It contains medical data of nearly 40,000 adults and 8,000 newborns in ICUs. The median age of adult patients was 65.8 years, of which 55.9% were males and 11.5% were hospitalized. The database is mainly used for academic and industrial research, offering a variety forms of data in ICU including demographic characteristics, vital signs, experimental testing, diagnosis, dosage of drugs, length of stay and other critical care unit data. We use support vector machines (SVM) [22] to fit the physiological measured values at different measurement times. After preprocessing, we take 10 minutes as the frequency of time series from admission time to discharge time. Please refer to [19] for more details in data processing.

### Formulation of the MDP

Following previous studies, the decision-making problem is modeled as an MDP by a tuple of <*S*,*A*,*P*,*R*>, where *s*_*t*_∈*S* is a patient’s state at time *t*, *a*_*t*_∈*A* is the action made by clinicians at time *t*, *P*(*s*_*t*+1_|*s*_*t*_,*a*_*t*_) is the probability of the next state after given the current state and action, and *r*(*s*_*t*_,*a*_*t*_)∈*R* is the observed reward following a transition at time step *t*. The goal of an RL agent is to learn a policy to maximize the expected accumulated reward over time horizon *T* by:

$$R^{\pi}\left(s_{t}\right) = \lim\limits_{T \to \infty} E_{s_{t+1}|s_{t}, \pi\left(s_{t}\right)} \sum \limits_{t+1}^{T} {\gamma}^{t} r\left(s_{t}, a_{t}\right) $$

where the discount factor *γ* determines the relative weight of immediate and long-term rewards.

The MDP of ventilation and sedation dosing in ICUs can be express as follow.

**State:** A patient’s state is composed of 13-dimensional features, including respiration rate, heart rate, arterial pH, positive end-expiratory pressure (PEEP) set, oxygen saturation pulse oxymetry (SpO2), inspired oxygen fraction (FiO2), arterial oxygen partial pressure, plateau pressure, average airway pressure, mean non-invasive blood pressure, body weight (kg) and age.

**Action:** The two discrete actions regarding ventilation are defined as whether weaning off a patient from the ventilator. As for the sedative, the propofol was discretized into four different actions. Ultimately, there are eight action combinations.

**Reward:** The reward function *r*_*t*+1_ is defined as $r_{t+1} = r_{t+1}^{vitals}+r_{t+1}^{vent\ off}+r_{t+1}^{vent\ on}$ [16, 19], in which $r_{t+1}^{vitals}$ evaluates the effect of these actions on the physiological stability of the patient within a reasonable range, $r_{t+1}^{vent\ off}$ estimates the performance of ventilation being stopped at time *t*+1, and $r_{t+1}^{vent\ on}$ simply represents the cost per hour on the ventilator.

## Methods

Machine learning can be divided into three categories: supervised learning, unsupervised learning and RL. Supervised learning continuously reduces the error between the predicted value and the original value by the tagged data. The common problems of supervised learning application are classification and regression. Unsupervised learning, however, aims at finding correlation of data without labels in clustering and dimension reduction. Unlike traditional supervised learning methods that usually rely on one-shot, exhaustive and supervised reward signals, RL tackles with sequential decision making problems with sampled, evaluative and delayed feedback simultaneously [2]. The sequential decision making process of medical problems usually includes multiple steps in sequence, and RL is good at dealing with such problems. We can build an MDP model and use an RL algorithm to learn an optimal treatment strategy. The long-term goal of RL is to maximize the cumulative reward value, which means that patients must recover, but there is also a risk of drug use that deviates from clinician guidance. Therefore, we incorporate clinician guidance in the framework of RL such that the action of drug selection is in line with the guidance of clinicians.

### Algorithm principle

**Fig. 1:**
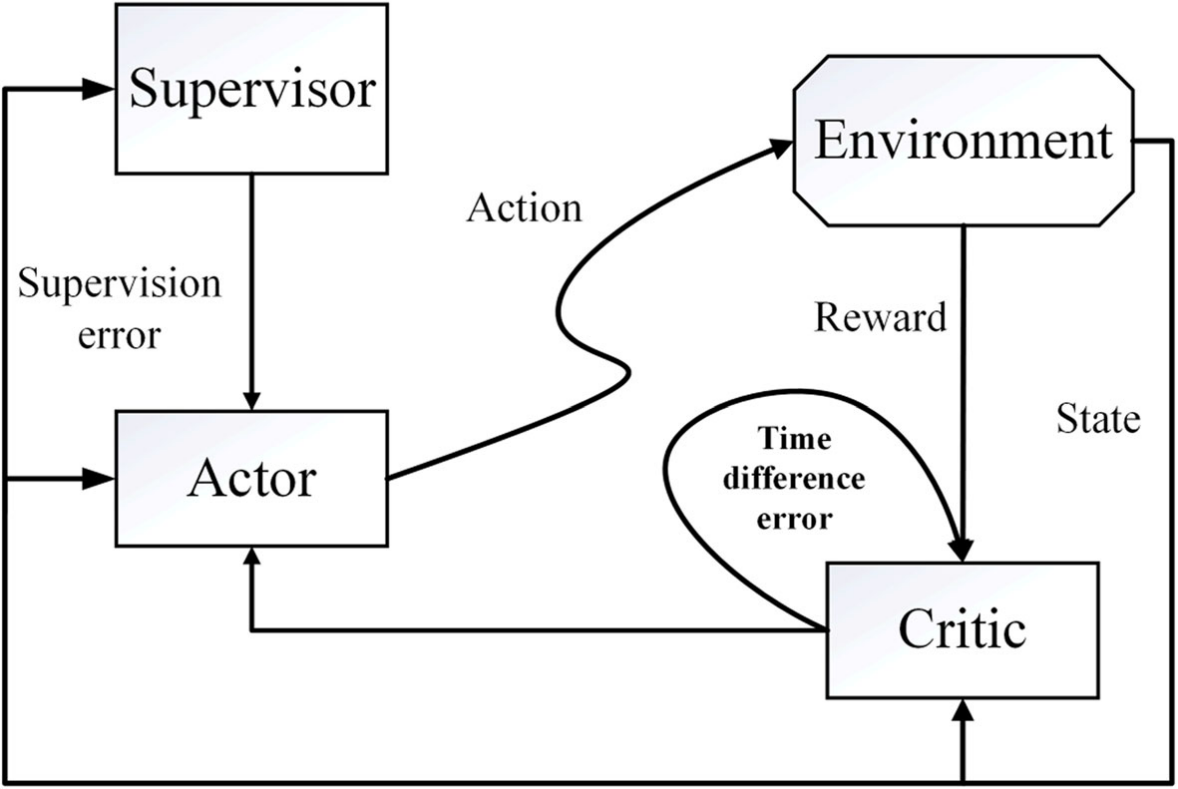
The framework of SAC algorithm

The framework of SAC algorithm is shown in Fig. 1. Based on AC, a supervised learning mode (Supervisor) is added to the Actor network, which changes the gradient direction and updates the hyperparameters of the Actor network [23]. During the Actor network optimization, the Supervisor is optimized at the same time. The Critic computes the value functions based on the Reward and State in current Environment, then passes the TD error to the Actor. The Actor updates the strategy based on the Critic’s TD error and the supervision error from the Supervisor.

### Actor network update

The Actor network obtains the best strategy by updating hyper-parameters *θ*. The input of this network is state and the output is action. We use the TD error and supervised learning error to optimize hyper-parameter *θ*. To this end, we propose the following formula:

$$J(\theta) = (1-\epsilon)J_{RL}(\theta)+\epsilon\left(-J_{SL}(\theta)\right) $$

where *J*_*RL*_(*θ*) represents the optimization goal of an RL algorithm, which represents the reward value expectation of the trajectory under the current strategy, and *J*_*SL*_(*θ*) is the optimization goal of supervised learning, which represents the degree of difference between the predicted action and the labeled action. *J*_*SL*_(*θ*) is usually expressed in the form of variance or conditional entropy between the predicted value and the original value. *ε* is a weighting parameter to balance the contribution between RL and supervised learning.

Our goal is to maximize the reward value and make less difference in the actions between the RL agent’s actions and the clinician’s actions in the process of optimizing the strategy. We use the method of stochastic gradient descent [24] to optimize the parameter *θ* as follows:

$$\theta = \theta+\alpha\left((1-\epsilon) \frac{\partial J_{BL}(\theta)}{\partial \theta} + \epsilon\left(-\frac{\partial J_{SL}(\theta)}{\partial \theta}\right)\right) $$

where *α* represents the learning rate.

### Critic network update

The Critic network guides the learning of the Actor network, while the Actor network outputs the final treatment strategy. The Critic network estimates the action-state reward value *Q*_*w*_(*s*,*a*). During learning, the Critic network outputs a predicted *Q* value *Q*(*s*_*t*_,*a*_*t*_) through *Q*_*w*_(*s*,*a*). The update of the Critic network parameter *θ* is as follows:

$$J(w) = E_{r_{t}, s_{t}}\left[\left(Q_{w}(s_{t}, a_{t})-y^{t}\right)^{2}\right] $$

$$y_{t} = r\left(s_{t}, a_{t}\right)+\gamma Q_{w}^{tar}\left(s_{t+1}, \mu_{\theta}(s_{t+1})\right) $$

where *J*(*w*) is the loss function of the Critic network, and $Q_{w}^{tar}$ is the target network parameter of the Critic network.

## Results

### Experimental setup

Since the feature is not always continuous and it may be a classification value, it is meaningless to compare such values. For example, [Red, Yellow, Blue] can be mapped to [0, 1, 2] to reflect their relationships but this mapping does not capture the relationship within the original feature attributes. This problem can be solved by using a one-hot encoding by using an *N*-bit status register to encode the *N* states, each state having its own separate register bit, and only one bit is active at any time [25]. Taking the above problem as an example, after using the one-hot encoding, [red, yellow, blue] can be encoded as [[0,0,1],[0,1,0],[1,0,0]] so that the original relationship of features can be maintained. In our formulation, the actions for the ventilation and sedative doses are encoded using the one-hot encoding, separately.

In the MDP of ventilation, the state is the 13-dimensional patient’s physiological characteristics, ventilator parameters, and current ventilation status. In AC and SAC, the Actor network, the Critic network and the Supervisor network all adopt a three-layer neural network. The Actor network has 20 neurons in the hidden layer with the ReLU activation function, and two neurons in the output layer with Softmax as the activation function. The Critic network has 20 neurons in the hidden layer, using the ReLU activation function, and a neuron in the output layer without using an activation function. The Supervisor network has 9 neurons in the hidden layer and two neurons in the output layer, and the Softmax is also used as the activation function for the output layer.

In the MDP model of sedative dose, the state is a 14-dimensional feature of the previous 13-dimensional feature combined with the ventilation action. The action is a sedative dose, which is encoded by the One-Hot encoding to form a four-category option. The network structure of it is the same as the MDP model of ventilation.

### Experimental results and analysis

**Fig. 2:**
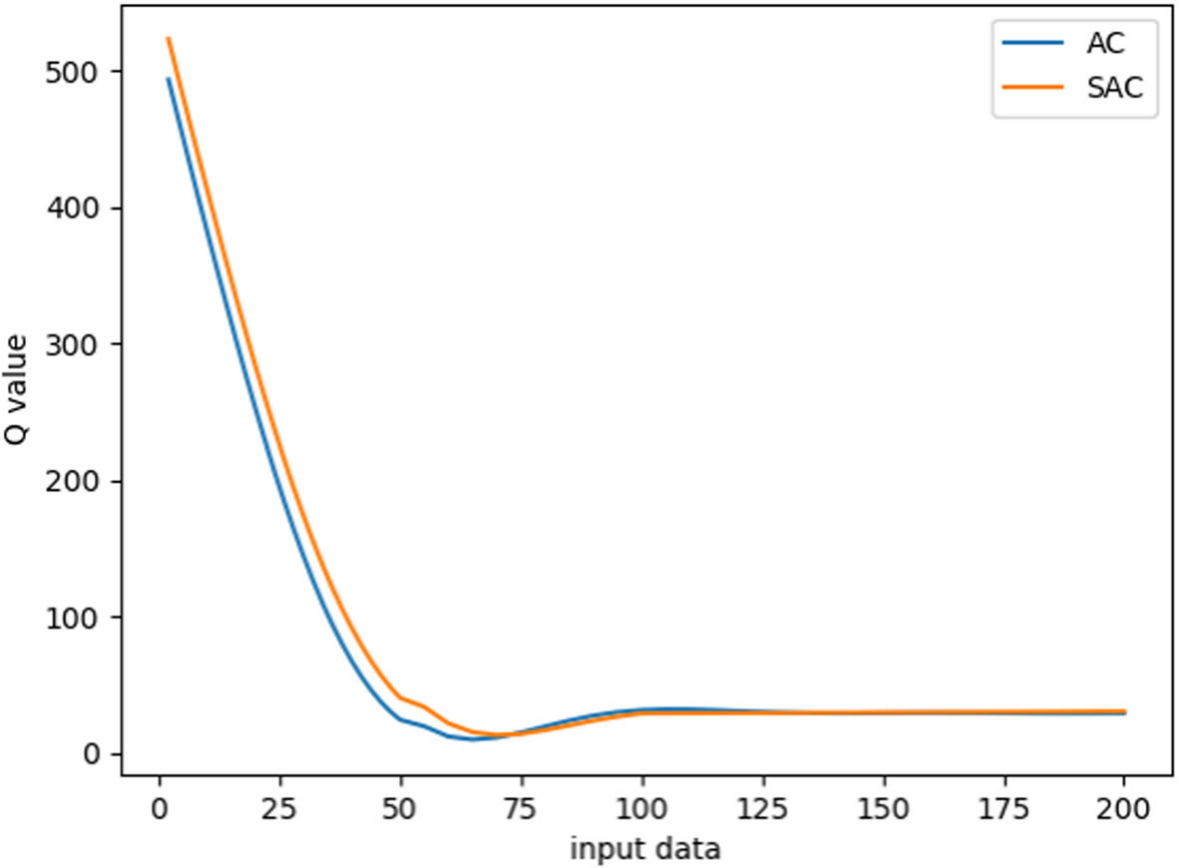
Learning dynamics in terms of Q values regarding ventilation using SAC and AC algorithms

**Fig. 3:**
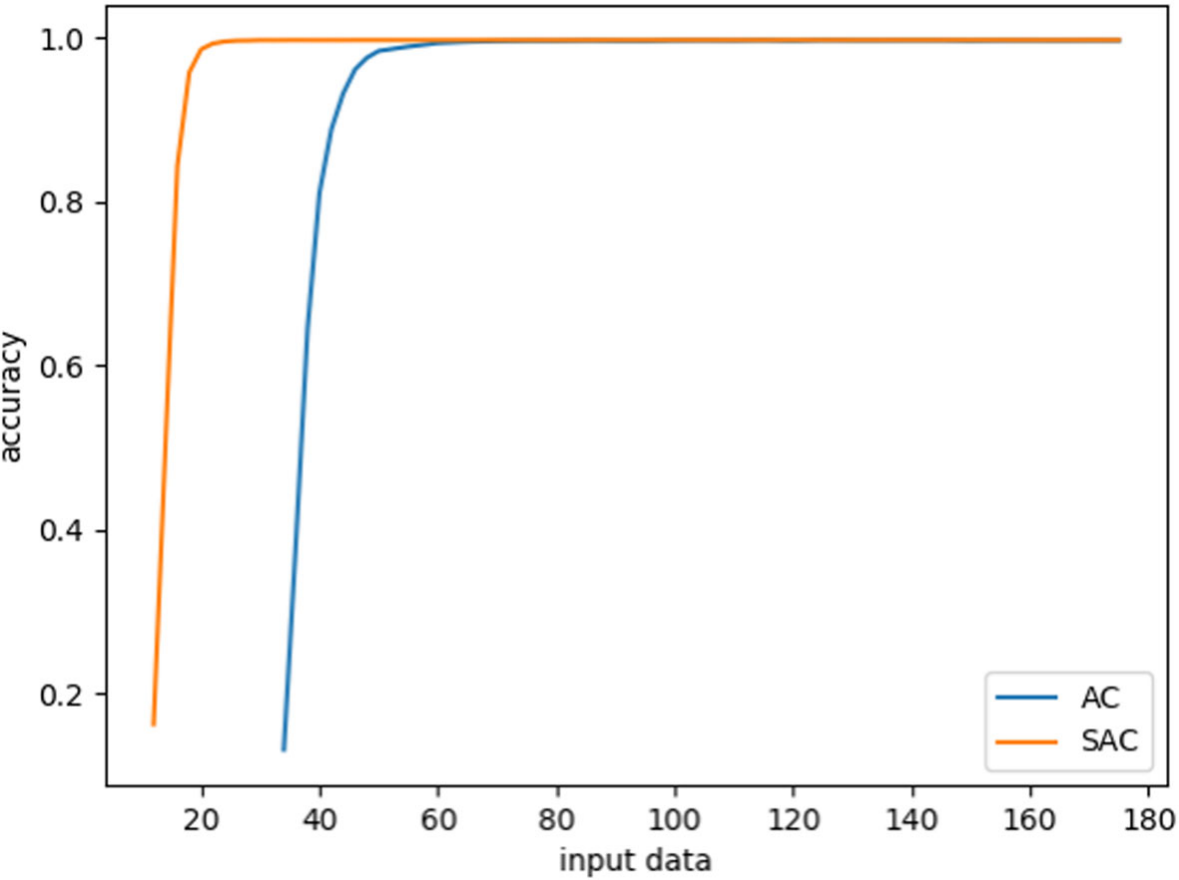
The process of SAC and AC algorithms evaluating the accuracy of the test set on ventilation

Figure 2 shows the results in the ventilation experiment, where the vertical axis is the Q value of the sample data, and the horizontal axis represents the training process. As the training proceeds, the Q value decreases gradually. Finally, both SAC and AC converge and reach a stable level. The trend of decline is basically the same, illustrating that both SAC and AC have a similar network structure for Q value prediction. However, SAC converges a bit faster than AC from a slightly higher initial values. Figure 3 shows that the Accuracy rate (AR) of the two algorithms has increased significantly with the increasing of episodes, and the stability level is above 95%. However, it can be seen that the convergence speed of the SAC algorithm is much faster than the AC algorithm. It takes 20 episodes for SAC to converge to 99% AR, and 60 episodes for AC. This is due to the Supervisor network in SAC, which can update the Actor network with simultaneous guidance of both the Supervisor part and the Critic part.

To further validate the effectiveness of SAC, we test the learned policy on the testing sets of expert and non-expert data sets. As shown in Table 1, both SAC and AC algorithms have an AR of over 99% on the testing set. The AR of SAC is slightly higher than that of AC, which indicates that the strategy learned by SAC is closer to the real medical strategy than that by AC. Meanwhile, it is worth noting that SAC is more accurate on expert data sets than on other data sets, while the AR of AC algorithm is comparatively balanced on each data set. It shows that the strategy learned by SAC is closer to the strategic plan of the experts.

**Table 1:** The AR of learned strategies using SAC and AC algorithms on the test data set

Strategy	Validation set	Expert data	Common single intubation	Multiple intubation
SAC	99.55%	99.57%	99.51%	99.55%
AC	99.48%	99.47%	99.46%	99.49%

**Fig. 4:**
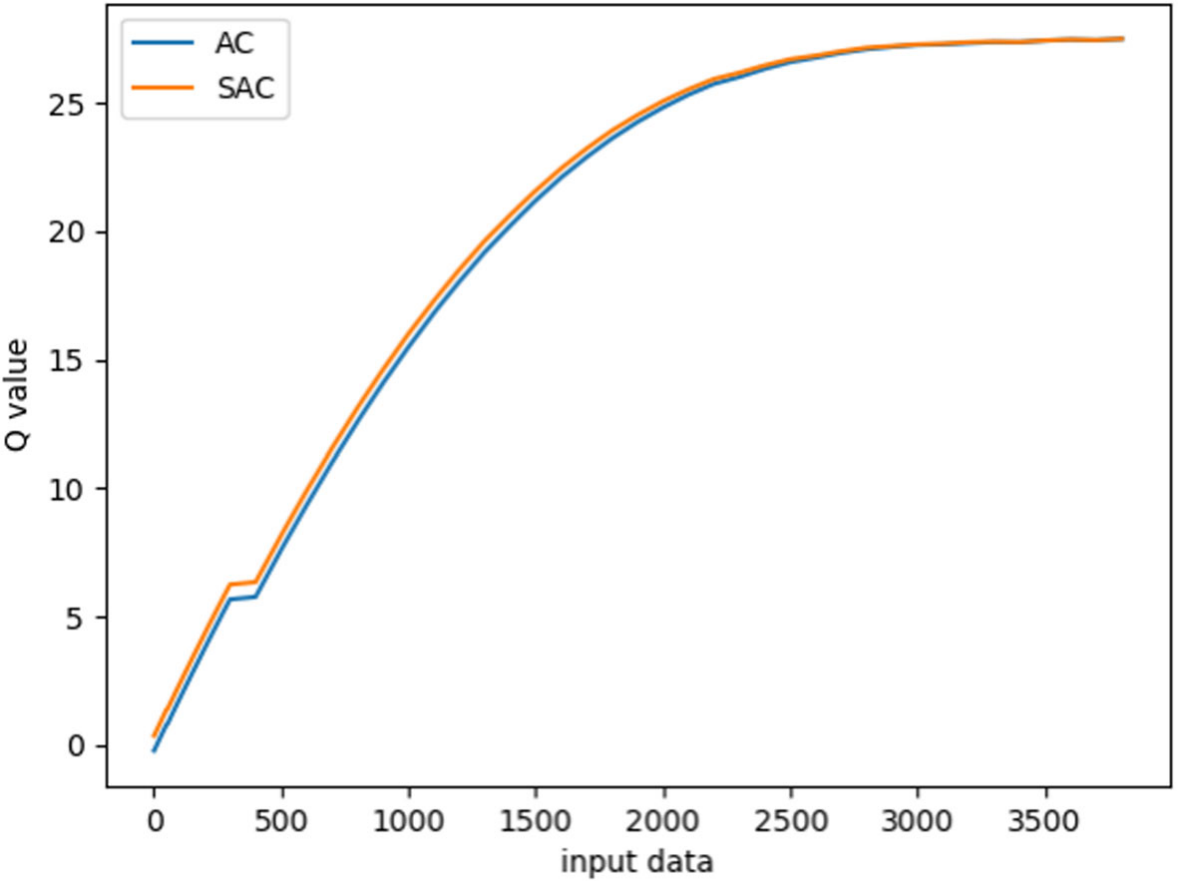
Learning dynamics in terms of Q values regarding sedative using SAC and AC algorithms

**Fig. 5:**
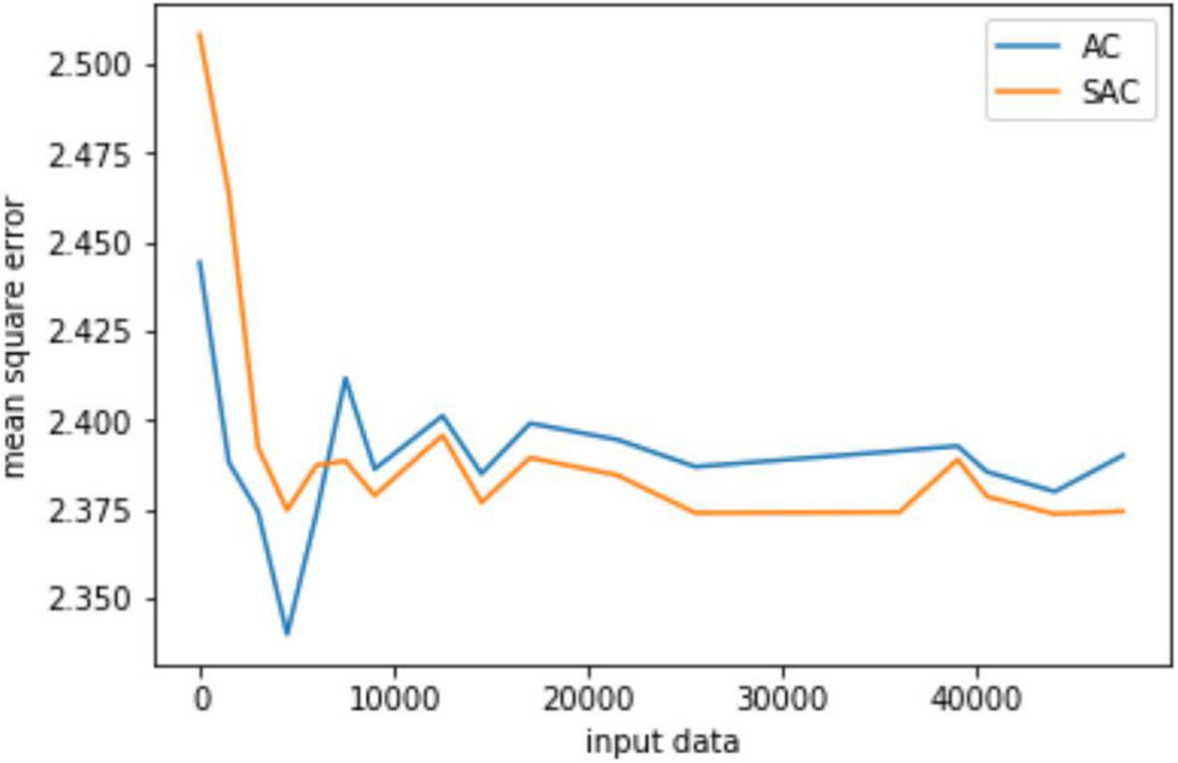
MSE reduction process of SAC and AC algorithms on the train set for sedative dosing

Figure 4 gives the experiment results for the sedative dosing. Since the learning data and the network structure are consistent during the process of ventilation experiment, the convergence trend of the Q value of SAC and AC algorithms is roughly the same. Figure 5 shows the mean square error (MSE) between the predicted and original values of the sedative dose on the train set. It can be seen that both SAC and AC can converge to a stable MSE after 10000 episodes. However, after that, the convergence value of SAC is lower than that of AC, which indicates that the strategy learned by SAC is closer to the clinician’s treatment strategy than that by AC. Besides, SAC is relatively more stable than AC in the final episode. This is because SAC introduces a supervised learning process such that a higher deviation from the clinician’s strategy can be reduced to a smaller value. The AC algorithm without supervision network only aims at maximizing the cumulative reward value, causing great fluctuations in the learning process.

## Discussions

We further analyze the differences between MSE and AR of two algorithms on the test set as shown in Table 2. In terms of AR, the SAC algorithm is slightly better than the AC algorithm, indicating that the strategy learned by SAC is closer to the real medical strategy than that of the AC algorithm. Although SAC and AC have the same AR on test set, SAC has a smaller MSE than AC. Thus, the SAC algorithm is closer to the real treatment strategy than the AC algorithm under the same AR. Table 3 shows the performance of two algorithms in expert data, single intubation and multiple intubation data. The performance of SAC is better than AC in MSE and AR on expert data set and non-expert data set. Especially, in multiple intubation data set, the AR of SAC is 6% higher than AC, and the MSE is reduced by 0.8. This illustrates that the goal of the clinician is indeed to cure the patient, but it is necessary to maintain a stable state of the patient under complex medical environments. Therefore, the introduction of supervised RL is more in line with the medical settings than the simple RL alone.

**Table 2:** The AR and MSE of learned polices using SAC and AC algorithms on the test data set

Strategy	MSE	AR
SAC	2.49	41.5%
AC	3.10	41.5%

**Table 3:** The AR and MSE of learned polices using SAC and AC algorithms on expert data, single intubation and multiple intubation

Strategy	Expert data		Single intubation		Multiple intubation	
	MSE	AR	MSE	AR	MSE	AR
SAC	2.52	37%	2.71	38%	2.28	47%
AC	3.01	34%	3.15	35%	3.08	41%

## Conclusions

In this paper, we first introduce the principle and advantages of incorporating supervised learning into RL, and then establish the MDP for mechanical ventilation and the dose of sedatives for patients in ICUs. During the process of learning the strategies, SAC not only achieve the long-term goal of curing patients, but also meet the short-term goal of approaching the clinician’s strategy gradually. Compared with the AC algorithm, SAC is more suitable to solve the problem of ventilation and the dose of sedatives in ICUs. Finally, we validate that SAC algorithm is slightly better than the AC algorithm in matching the clinician strategy, and its convergence speed and data utilization efficiency are much higher than AC. In the future, we will apply the SAC algorithm to other healthcare domains such as HIV and Sepsis to achieve more efficient and stable dynamic treatment regimes.

## Data Availability

The datasets used and/or analysed during the current study is available from the first author on reasonable request.

## Abbreviations

RL: Reinforcement learning
ICUs: Intensive care units
HIV: Human immunodeficiency viruses
SBTs: Spontaneous breathing tests
MIMIC: Medical information mart for intensive care
MIT: Massachusetts Institute of Technology
BIDMC: Beth Israel Dikang Medical Center
SVM: Support vector machine
MDP: Markov decision process
AC: Actor-critic
SAC: Supervised actor-critic
AR: Accuracy rate
MSE: Mean square error
